# Macrophage TGF-*β*1 and the Proapoptotic Extracellular Matrix Protein BIGH3 Induce Renal Cell Apoptosis in Prediabetic and Diabetic Conditions

**DOI:** 10.4236/ijcm.2016.77055

**Published:** 2016-07-21

**Authors:** Robert J. Moritz, Richard G. LeBaron, Clyde F. Phelix, Rajesha Rupaimoole, Hong Seok Kim, Andrew Tsin, Reto Asmis

**Affiliations:** 1 Department of Biology, University of Texas at San Antonio, San Antonio, USA; 2 Departments of Biochemistry and Clinical Laboratory Sciences, School of Health Professions, University of Texas Health Science Center at San Antonio, San Antonio, USA

**Keywords:** TGF, BIGH3, Macrophages, Diabetes, Extracellular Matrix

## Abstract

Metabolically stressed kidney is in part characterized by infiltrating macrophages and macrophage-derived TGF-*β*1 that promote the synthesis of various ECM molecules. TGF-*β*1 strongly enhances the expression of the gene *TGFBI* that encodes a cell-adhesion class, proapoptotic ECM protein called BIGH3. We hypothesized that in a diabetic environment a relationship between infiltrating macrophages, macrophage-derived TGF-*β*1, and BIGH3 protein promotes renal cell death. To investigate this hypothesis, we used our mouse model of diabetic complications. Mice on a high-fat diet developed hypercholesterolemia, and exposure to streptozotocin rendered hypercholesterolemic mice diabetic. Immunohistochemical images show increased macrophage infiltration and BIGH3 protein in the kidney cortices of hypercholesterolemic and diabetic mice. Macrophages induced a two-fold increase in BIGH3 expression and an 86% increase in renal proximal tubule epithelial cell apoptosis. TGF-*β*1 antibody and TGF-*β*1 receptor chemical antagonist blocked macrophage-induced apoptosis. BIGH3 antibody completely blocked apoptosis that was induced by TGF-*β*1, and blocked apoptosis induced by exogenous recombinant BIGH3. These results uncover a distinctive interplay of macrophage-derived TGF-*β*1, BIGH3 protein, and apoptosis, and indicate that BIGH3 is central in a novel pathway that promotes diabetic nephropathy. Macrophage TGF-*β*1 and BIGH3 are identified as prediabetic biomarkers, and potential therapeutic targets for intervention in prediabetic and diabetic individuals.

## 1. Introduction

Advances in clinical treatments and strong emphasis on diabetes education have played significant roles in prediabetic and diabetic health care management. However, a continued increase in the number of clinical cases of diabetic complications has increased in recent years. Moreover, a greater percentage of minority individuals experience clinical level diabetic diseases of the renal, vascular, and ocular systems when compared to individuals in the non-minority population, highlighting a health disparity element in diabetes [1]. When poorly controlled, diabetes mellitus types I and II promote harmful complications in targeted organs and tissues, including the renal system. Even with modern therapeutic interventions, dysregulated cell signaling, changes in ECM turnover, and apoptosis promote kidney damage and end-stage renal disease, highlighting a need for a more complete understanding of the mechanisms underlying diabetic nephropathy. Development of diabetic nephropathy generally involves monocyte/macrophage infiltration, an increase in the local production of cytokines and reactive oxygen species (ROS), increased apoptosis, and accumulation of ECM molecules. The latter is explained, in part, by cytokine-induced changes in the expression of genes that encode for the ECM molecules fibronectin, collagen types I and IV, laminin and proteoglycans [2]–[4]. TGF-*β*1 activates the gene *TGFBI*, which encodes the ECM molecule called BIGH3 (Transforming Growth Factor BInduced Gene Human Clone 3), also known as beta-ig, TGFBI, and kerato-epithelin. BIGH3 is a cell adhesion-class protein that comprises a cysteine-rich domain, four fasciclin-1 (FAS1)-like repeats, and in the C-terminal portion EPDIM and RGD integrin-binding sequences [5] [6]. In its secreted full-length form (minus the secretory sequence) SDS PAGE analysis estimates a mass of 69 kDa. The C-terminal portion of the 69-kDa BIGH3 becomes cleaved, yielding a stable 60-kDa BIGH3 protein, and generating C-terminally derived integrin-ligand peptides that appear to act as competitive inhibitors of the pro-survival ECM-integrin interactions [7]–[9]. These BIGH3-derived C-terminal peptides induce apoptosis, giving rise to the term BMA (BIGH3-mediated apoptosis), which we use in this study. Several different laboratories have reported BMA on retinal pericytes [10], retinal endothelial cells [11] the osteosarcoma cell lines MG-63 and Saos-2 [9], transformed corneal epithelial cells [12], Chinese hamster ovary cells, and other cell types [8], thus the rationale to classify BIGH3 as a proapoptotic ECM protein.

Although macrophages play important roles in tissue homeostasis, they are also major contributors to diabetic progression, promoting metabolic stress and interstitial fibrosis, leading to loss of renal function [13] [14]. In rodents, blocking macrophage infiltration attenuated kidney tubular epithelial cell apoptosis [15]. Similarly, deleting *CCL*2, which encodes monocyte chemoattractant protein-1, attenuated renal tubule cell damage [16], and suppressed development of diabetic nephropathy in streptozotocin (STZ)-treated mice [17]. Macrophage-derived soluble molecules, *i.e.* NO, ROS, have been implicated in rodent glomerular mesangial cell apoptosis [18]. Macrophage ingestion of apoptotic bodies *in vitro* induce macrophages to secrete TGF-*β*1 [19] [20], accounting for at least some of the TGF-*β*1 in tubulointerstitium and glomeruli of diabetic patients [21]. These same loci, *i.e.*, tubulointerstitium and glomeruli, in adult diabetic rat show an increase in BIGH3 protein [22] [23], suggesting that in diabetic humans and animals, macrophage-derived TGF-*β*1 stimulates BIGH3 protein synthesis. Here we document a previously unrecognized relationship involving macrophages, macrophage-derived TGF-*β*1, an increase in BIGH3 synthesis, and an increase in BMA of renal cells. Discussed is a potential positive feedback mechanism that promotes BMA and diabetic nephropathy.

## 2. Methods

### Animals

Female LDL-R^−/−^ mice (B6.129S7-LDLR^tmIHer^/J, stock no. 002207) on a C57BL/6J background and C57BL/6J donor mice were obtained from The Jackson Laboratory (Bar Harbor, ME). To render mice diabetic, STZ dissolved in 50 mM citrate buffer (pH = 4.5), was used for intraperitoneal injection at a dose of 50 mg·kg^−1^·day^−1^ for five consecutive days and, after two days rest, again for two consecutive days [24] [25]. Non-diabetic mice received a comparable volume of citrate buffer. To induce hypercholesterolemia, diabetic mice were fed a high fat diet (HFD), BioServ AIN-76A, fat: 21% wt/wt; cholesterol: 0.15% wt/wt (Frenchtown, NJ) plus STZ for a total of 12 weeks beginning 3 weeks after the first STZ injection. Non-diabetic mice received either a MD (low-fat maintenance diet, BioServ, AIN-93G) or a HFD. All mice were 17 weeks of age and 18.4 ± 0.2 g at the beginning of the study.

Survival rates of STZ treated female mice significantly exceeded that of corresponding STZ treated male mice, female mice were selected for this study. All studies were performed with the approval of the University of Texas Health Science Center at San Antonio Institutional Animal Care and Use Committee.

### Renal Cell Culture

Human renal proximal tubule epithelial cells (RPTEC), renal epithelial basal medium supplements (human epidermal growth factor, hydrocortisone, epinephrine, insulin, triiodothyronine), and the antibiotic and anti-fungal agents (gentamicin sulfate and amphotericin B) were purchased from Clonetics/Lonza (Allendale, NJ), and ATCC. These supplements combined with 10% FBS constituted renal epithelial growth medium (REGM). RPTEC monolayers that were 70% confluent were used to initiate subcultures at 5 × 10^4^ cells per ml medium. All incubations of live cells, for maintenance and experiments, occurred in a humidified 37°C incubator saturated with 5% CO_2_ in 95% ambient air. All experiments were conducted using RPTEC at or below passage eight.

### Macrophage-conditioned Media

Mononuclear cells were isolated from blood obtained from healthy human donors through the South Texas Blood and Tissue Center (San Antonio, TX). Mature human monocyte-derived macrophages (HMDM) were prepared as described previously [26]. Two different conditioned media were generated for this study. To mimic normal “healthy” conditions, mature HMDMs were cultured for 24 hours in standard RPMI medium, followed by rinsing and a 24-hour incubation in serum-free RPMI medium. This serum-free conditioned medium was designated macrophage-conditioned medium (MCM). To generate a “diabetic” environment, we cultured mature HMDMs in RPMI supplemented with 20 mM glucose (25 mM final glucose concentration) and 100 µg/ml freshly isolated human LDL. After 24 hours the medium was removed, macrophage monolayers were rinsed and given serum-free RPMI for a 24-hour incubation period. This medium was designated diabetic macrophage-conditioned medium (dMCM). To remove any floating cells the media were centrifuged. Supernatants were kept at −20°C until use. TGF-*β*1 in the MCMs was quantified using Quantikine ELISA reagents following the manufacturer’s recommendations (R&D Systems). Briefly, 0.1 ml from each batch of MCMs with and without acid activation of latent TGF-*β*1 was used to quantify active and total TGF-*β*1. A standard active TGF-*β*1 curve was generated at the time of assessment.

### Antibodies

New Zealand white rabbits immunized with full-length recombinant BIGH3 provided antiserum that we have previously characterized [27]. Preimmunized rabbit serum was used as negative controls where indicated in the individual experiments. Anti-CD68 antibody, clone FA-11, and anti-TGF-*β*1 antibody, clone 9016, were from AbDSerotech (Raleigh, NC) and R&D Systems (Minneapolis, MN) respectively. Heating serum to 52°C for 10 min inactivated complement before use in experiments.

### Immunohistochemistry

Kidneys from adult MD, HFD and diabetic mice were harvested, immediately frozen in optimal cutting temperature compound [24] and stored at −80°C until use. From kidney cortex, 10-µm thick sections were fixed for 30 min in 10% fresh paraformaldehyde in PBS. Fixed sections were rinsed with 20 mM glycine in PBS (Buffer A), blocked for 1 hour in 3% BSA in PBS, and incubated 12 hours in Buffer A plus anti-CD68 antibody (1:300) and anti-BIGH3 antiserum (1:200), or with control serums. After rinsing, sections were incubated for 1 hour in blocking buffer with anti-rabbit or anti-mouse antibodies conjugated to DyLight488 and DyLight594 fluorescent dyes, respectively, then rinsed with Buffer A and sealed in Vectashield (Vector Laboratories, Burlingame, CA). Unless indicated otherwise, all incubations were at ambient temperature, and all rinses were 3 × 15 min in Buffer A.

### Recombinant BIGH3

Spodopterafrugiperda (Sf9) insect cells, GeneJuice transfection reagent and pIEX-3 Ek/LIC were from Novagen (Darmstadt, Germany). Sf9 cells were propagated in 30 ml of 28°C BacVector insect cell medium in 250 ml flasks in 140-rev/min motion. Sf9 cells were transfected with plasmid encoding a 6His-tagged human BIGH3 cDNA. Two days after transfection, the conditioned medium was exchanged to 10 mM imidazole in 0.3 M NaCl, 50 mM sodium phosphate buffer, pH 8 (Buffer B) using a 30-kDa cutoff membrane in a flow-cell apparatus. The retentate was applied over a 0.3 ml bed volume of Ni-NTA affinity resin that was subsequently washed with Buffer B. Increasing imidazole from 10 mM to 250 mM in Buffer B eluted BIGH3. Western blots identified the fractions containing BIGH3, which were pooled, and Buffer B exchanged for PBS using a 30-kDa cutoff Centricon concentrator. BIGH3 protein was quantified using SDS-PAGE gels, Western blots and bicinchoninic acid protein assays.

### Apoptosis Assays

Wells of an 8-well chamber slide, each seeded with 10^4^ RPTEC in REGM, were incubated 24 hours. Following REGM aspiration the wells received a mixture of REGM plus 25% dMCM, REGM plus 25% MCM, REGM plus BIGH3, or REGM only. After another 24-hour incubation, media were aspirated and the cells fixed in 4% paraformaldehyde in PBS, washed, and treated with 0.1% Triton X-100 before TUNEL assays (Roche Applied Science, Indianapolis, IN). After washing, Vectashield and coverslips were applied. The numbers of stained cells in ten different random fields at 20× magnification were counted using a ZEISS (Jena, Germany) Axioplan II fluorescent microscope. For ssDNA assays, BIGH3 was added to microtiter wells containing 10^4^ RPTEC in 0.1 ml 37°C REGM. After a 24-hour incubation and 5-minute centrifugation, the medium in each well was replaced with 0.2 ml of 80% methanol in PBS. Wells were incubated at 25°C for 30 min, dried, and 0.05 ml formamide was added to each well before a 20-min incubation at 75°C. Detection of ssDNA was accomplished using a monoclonal antibody and apoptosis detection reagents (APT225, Millipore, Billerica, MA). Absorbance at 405 nm quantified ssDNA. DAPI-stained nuclei of BIGH3-treated and non-treated cells provided images for nuclear condensation comparison.

### Quantitative PCR

Total RNA isolated from RPTEC was used to synthesize BIGH3 cDNA in accordance with the manufacturer’s protocol using TaqMan Reverse Transcription Reagents (ThermoFisher Scientific,Waltham MA). The cDNA obtained from total RNA served as template for qPCR amplification using Power SYBR Green (Applied Biosystems/Life Technologies, Grand Island, NY). BIGH3 transcripts were amplified using the forward and reverse primer pair 5’-TGGACAGACCCTGGAAACTC-3’ and 5’-GTCTCCCTTCAGGACATCCA-3’ respectively. Real-time PCR was performed on an Applied Biosystems 7300 system using 40 cycles of denaturation (95°C for 15 s) and annealing/extension (60°C for 60 s). Ct values of measured fluorescence of BIGH3 gene amplicon elevating above a fixed threshold were recorded and normalized to cDNA generated from 18s rRNA transcripts. Relative measurements were quantified using the ΔΔCT method.

### Western blots

Proteins resolved on reducing 4% – 10% SDS PAGE gels and transferred to Immobilon-P membrane were incubated with anti-BIGH3 antiserum. BIGH3 antibody was detected using a second antibody conjugated to horseradish peroxidase and the substrate 3,3’-diaminobenzidine. Densitometry analysis was accomplished using Image J (http://imagej.nih.gov/ij/).

### Statistical analysis

For statistical analysis Student’s t-test, One-Way ANOVA, and Two-Way ANOVA were performed and P values of less than 0.05 were set as statistically significant. Post Hoc statistical analysis methods used Bonferroni multiple comparisons test and Newman-Keuls Multiple Comparison Test. Results are expressed as ±SEM of the number of experiments. Statistical analysis was performed using GraphPad Prism software.

## 3. Results

### Metabolic stress increases macrophage infiltration and BIGH3 protein in kidney cortex

**Figure 1:**
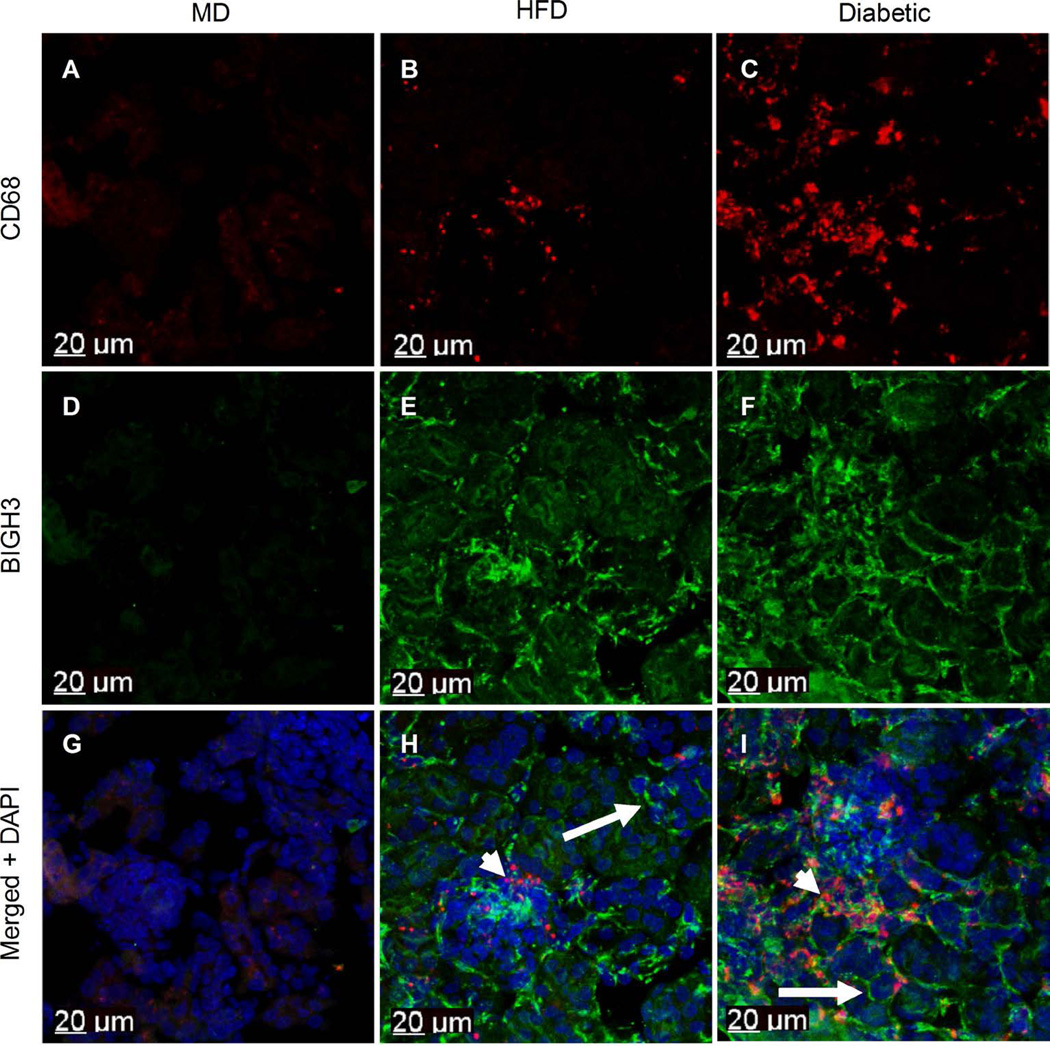
Metabolically-stressed kidney cortex show increased macrophage numbers and BIGH3 protein levels. Macrophages were stained with anti-CD68 antibody (red). BIGH3 antiserum staining and nuclei (DAPI) staining are green and blue, respectively. Images are from kidney cortices of healthy mice on a maintenance diet (MD) (A) (D) (G), a HFD (B) (E) (F) and from diabetic mice on a HFD (C) (F) (I). Short and long arrows indicate macrophages and BIGH3 protein respectively (H) (I). Scale bars (20 µm).

It is known that macrophages infiltrate diabetic kidney cortex, and that macrophage-derived TGF-*β*1 promotes ECM accumulation in diabetic cortical interstitium and basal lamina [28] [29]. TGF-*β*1 strongly upregulates expression of the BIGH3 gene, thus a logical expectation is that a greater quantity of macrophages and BIGH3 protein would be evident in kidney cortex of diabetic mice when compared to non-diabetic kidney cortex. To test this hypothesis, we utilized our previously characterized mouse model of diabetic complications [24]. Immunohistochemical analysis detected few, if any macrophages in the kidney cortex of healthy mice fed a MD (Figure 1(A)). In distinction, an increase in macrophage number was observed in the kidney cortex of HFD dyslipidemic mice (Figure 1(B)). When STZ rendered HFD mice diabetic, hereafter referred to as HFD + diabetic mice, a further increase in macrophage number was evident when compared to kidney cortices of mice on a MD and HFD (Figure 1(C)). Anti-BIGH3 antibody revealed little staining of BIGH3 protein in kidney cortex of mice on a MD (Figure 1(D)). Unexpectedly, BIGH3 staining was markedly increased throughout the cortex of mice on a HFD (Figure 1(E)), suggesting BIGH3 protein is a potential prediabetic biomarker (discussed below). Similarly, broad, extensive BIGH3 protein staining was evident in kidney cortex of HFD + diabetic mice (Figure 1(F)). In kidney cortices of mice on a HFD, and in HFD + diabetic mice, BIGH3 was observed at or near pericellular and interstitial matrices (Figure 1(H), Figure 1(I), long arrows) in greater quantities than in mice on a MD (Figure 1(G)). Also noted are macrophages at or near BIGH3 protein (Figure 1(H), Figure 1(I), short arrows), raising the question of whether BIGH3 serves as a substratum in macrophage recruitment and adhesion (see discussion). Here we conclude that HFD and diabetic conditions promote macrophage infiltration and BIGH3 protein synthesis in kidney cortices. We next investigated whether macrophage-derived soluble molecules increase BIGH3 gene expression.

### Macrophage-derived soluble molecules stimulate renal cells to synthesize BIGH3 transcripts

**Figure 2:**
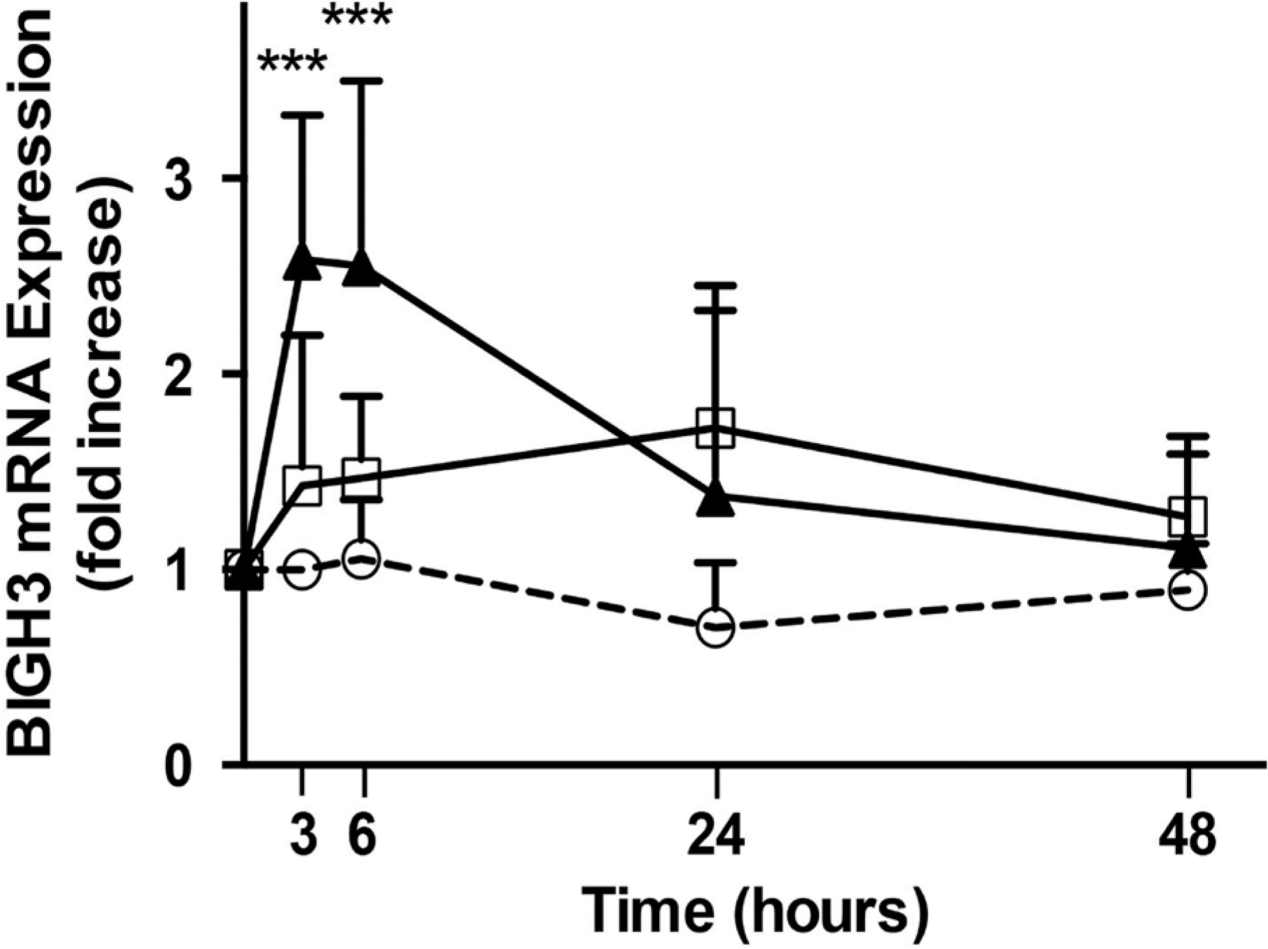
MCMs upregulate BIGH3 transcript expression. RPTEC (10^5^) cultured in REGM plus 25% RPMI (white circles), 25% MCM (white squares) or 25% dMCM (black triangles) express BIGH3 transcripts. At times indicated qPCR measured BIGH3 transcripts as described in the methods section. A Two Way ANOVA was performed for treatment and time. There were three treatments, RPMI, MCM, and dMCM; and there were 6 time points, 0, 3, 6, 12, 24, and 48 hours after exposure of RPTEC to the media. There were a total of 9 replicates in each group. Post hoc analysis for significant differences between groups was performed with a Bonferroni multiple comparisons test. Two Way ANOVA was significant [treatment: F (2, 120) = 26.22, p < 0.0001; time: F (4, 120) = 10.34, p < 0.0001; and interaction: F (8, 120) = 6.48, p < 0.0001]. Significance was set at p < 0.05, ***p < 0.001.

To examine whether macrophages produce factors that promote BIGH3 transcript expression in a diabetic environment, two different media were prepared; macrophage conditioned medium (MCM) and diabetic macrophage conditioned medium (dMCM). Briefly, macrophages were first pre-incubated for 24 hours with RPMI basal medium containing high (25 mM) glucose and high (100 µg/ml) LDL. This high glucose and LDL basal medium was aspirated, and macrophages were washed in RPMI and then used to condition serum-free RPMI for 24 hours to generate dMCM. Therefore, the dMCM used in experiments contained macrophage-derived soluble factors, but not high glucose or LDL. An identical procedure generated MCM, albeit macrophages were pre-incubated in RPMI basal medium without high glucose and LDL. Human RPTEC were maintained in REGM. For experiments, RPTEC were cultured for up to 48 hours in conditions where 25% of REGM was replaced with dMCM, MCM or RPMI. Results in Figure 2 show that dMCM induced an acute increase in BIGH3 transcript synthesis in RPTEC. Three hours post-exposure to dMCM, BIGH3 mRNA levels had increased 2.5 fold. RPTEC exposed to MCM increased BIGH3 mRNA synthesis initially. At 3 hours post-exposure to MCM, BIGH3 mRNA level decreased and then gradually increased 0.5-fold by 24 hours. In both conditions the increase in BIGH3 mRNA was transient, approaching the REGM/RPMI control baseline throughout the remaining 24 hours of the assay. REGM/RPMI did not significantly change BIGH3 transcript levels.

### RPTEC secrete and cleave BIGH3 protein

**Figure 3:**
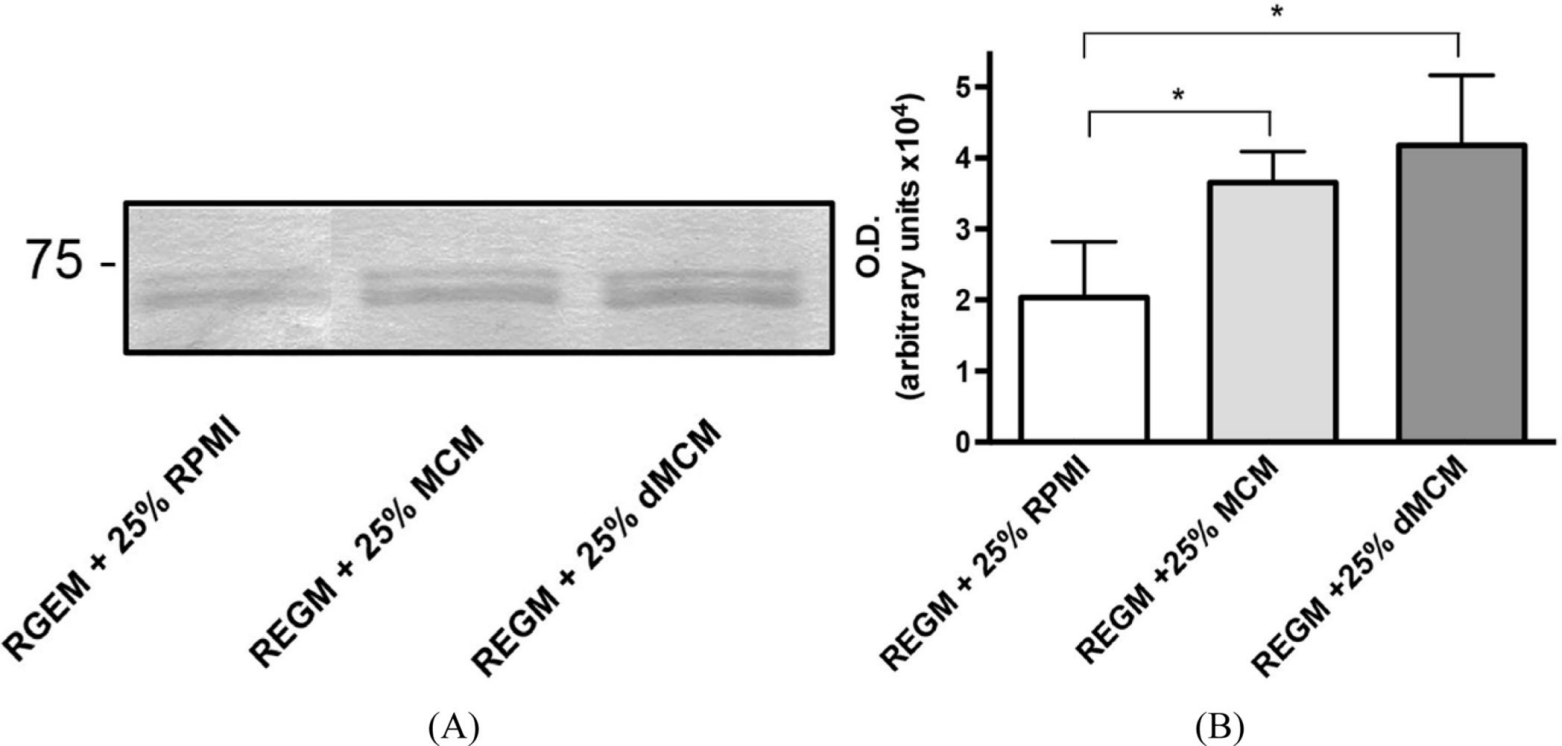
MCMs upregulate BIGH3 protein expression. (A) The differential mRNA levels agree with the quantity of BIGH3 protein in conditioned media. RPTEC (10^5^) were cultured in REGM plus 25% RPMI, MCM, or dMCM. After 48 hours incubation Western blots resolved proteins that were then stained with BIGH3 antibody. The upper bands are 69 kDa BIGH3 that has the intact C-terminus. The lower bands are C-terminally cleaved BIGH3. Protein loaded on SDS PAGE was normalized to total cellular protein. The position of the 75-kDa protein standard is indicated. (B) Densitometry quantified BIGH3 protein in each of the three culture conditions. One-Way ANOVA indicates that there is significantly more BIGH3 in MCM and dMCM compared to BIGH3 in RPMI (p < 0.05), but no significant differences were evident when comparing BIGH3 protein levels in MCM to dMCM.

Both dMCM and MCM induced greater quantities of secreted BIGH3 protein when compared to the BIGH3 protein in REGM/RPMI control medium (Figure 3(A), Figure 3(B)). BIGH3 protein increased with active TGF-*β*1 in MCM (41 – 53 pg/ml TGF-*β*1) and dMCM (60 – 76 pg/ml TGF-*β*1). Albeit TGF-*β*1 levels in dMCM were higher than levels in MCM, a significant increase was not apparent when comparing BIGH3 protein levels in MCM and dMCM (Figure 3(B)). This result suggests TGF-*β*1 levels in MCM saturated TGF-*β*1 receptor signaling. If true, then a higher TGF-*β*1 level in dMCM would not equate with additional BIGH3 synthesis. This suggests that in a prediabetic environment macrophage TGF-*β*1 increases BIGH3 levels close to BIGH3 levels in a diabetic environment, consistent with the idea that in a prediabetic environment BIGH3 protein in kidney cortex is already accumulating in the interstitial space (Figure 1(E)). Additional experiments are necessary to quantify the extent of differences in BIGH3 protein in prediabetic and diabetic kidney cortex. BIGH3 in MCM and dMCM, when compared to BIGH3 in control medium, were significant. Although fresh control medium did not contain detectable levels of TGF-*β*1, modest levels of BIGH3 transcripts (Figure 2) and BIGH3 protein (Figure 3), in the control medium indicates that RPTEC synthesize some of the BIGH3 in MCM and dMCM.

On SDS PAGE the relative mobility of secreted BIGH3 was calculated to be 69 kDa [9], which is the estimated size of the upper BIGH3 band detected with polyclonal anti-BIGH3 antibody. The antibody also detected BIGH3 protein at approximately 60 kDa (Figure 3(A)). These results agree with previous mass spectrometry analysis showing that BIGH3 protein is cleaved within its C-terminus, generating 62- and 60-kDa BIGH3 proteins, and Western blots showing the 62- and 60-kDa proteins as closely-spaced bands [7]–[9] [30]. We conclude that 1) macrophages in a HFD or diabetic environment provide soluble molecules that increase BIGH3 gene transcription and translation and 2) RPTEC cleave the C-terminus of BIGH3 to generate a 60-kDa mature protein and C-terminally-derived peptides. A logical hypothesis is that in MCM and dMCM, TGF-*β*1 is the soluble factor that increases BIGH3 gene transcription and translation, followed by BIGH3 secretion and BIGH3 C-terminal cleavage (see discussion).

### Blocking TGF-β1 receptor signaling inhibits BIGH3 transcript expression

**Figure 4:**
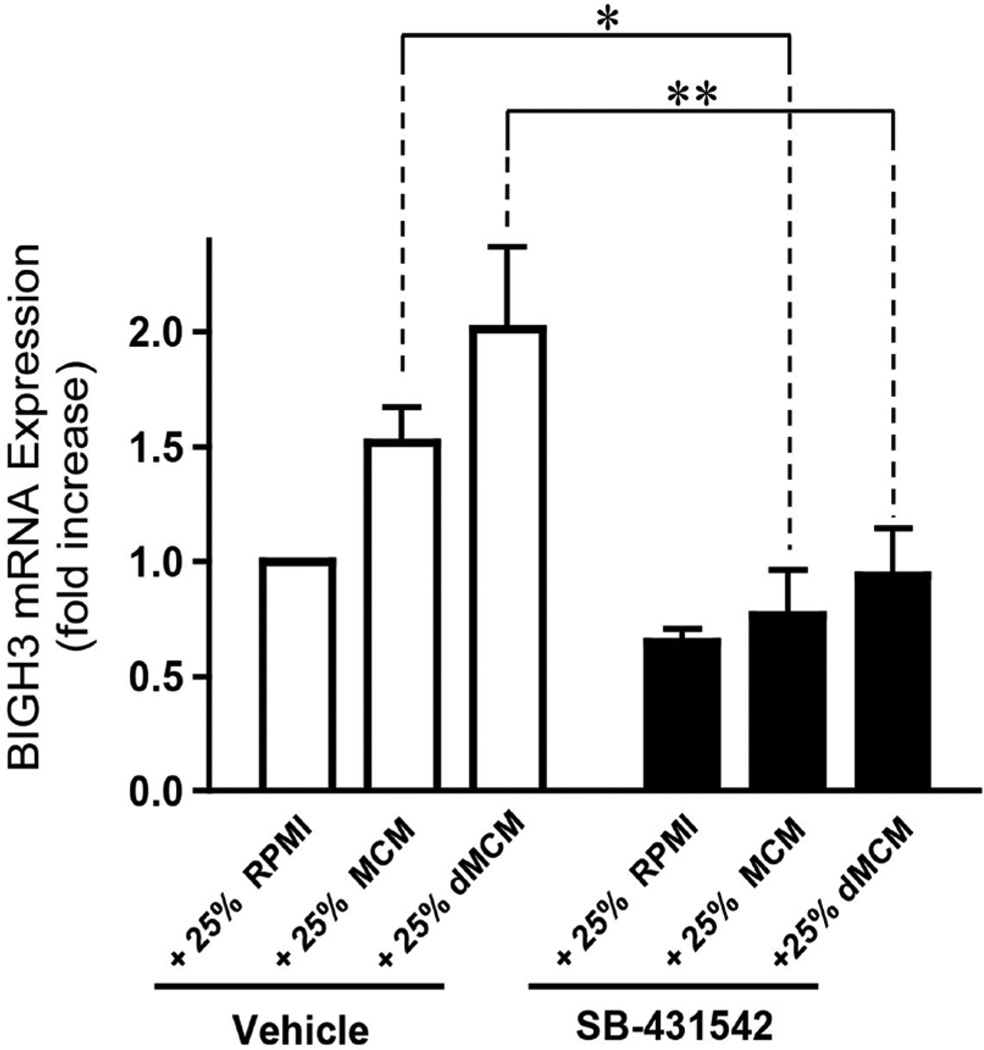
Macrophage-derived TGF-β1 upregulates BIGH3 transcript expression. Cells were cultured in REGM + 25% RPMI, REGM + 25% MCM and REGM + 25% dMCM, each with the TGF-β1 receptor inhibitor SB-431542 or DMSO vehicle. After a 24-hour incubation period qPCR measured BIGH3 transcripts. A Two Way ANOVA was performed with a Bonferroni Multiple Comparison post hoc test for significant differences. The Two Way ANOVA was significant for SB-431542, p < 0.0001, and for medium, p < 0.05. The effect of SB-431542 on MCM was significant at *p < 0.05 and on dMCM at **p < 0.01.

To test whether macrophage-derived TGF-*β*1 in dMCM and MCM induce BIGH3 gene transcription, we used the pharmaceutical small chemical inhibitor SB-431542 that selectively blocks TGF-*β* type I receptor kinase signaling. Results show that 13 nM SB-431542 blocked BIGH3 gene transcription by 50% and 53% respectively in REGM plus 25% dMCM, and REGM plus 25% MCM, when compared to vehicle only controls (Figure 4). These data show that TGF-*β*1 signaling in RPTEC is a strong transcriptional stimulator of the BIGH3 gene. In the control medium (no MCM/dMCM added), SB-431542 blocked BIGH3 expression by 30% (Figure 4). This result indicates that RPTEC themselves synthesize low-levels of TGF-*β*1 that induce some BIGH3 expression, as predicted in the previous results (Figure 2 and Figure 3). We conclude that macrophage-derived TGF-*β*1 and TGF-*β*1 receptor signaling induces BIGH3 gene transcription.

### TGF-β1 upregulates RPTEC BIGH3 protein synthesis and secretion

**Figure 5:**
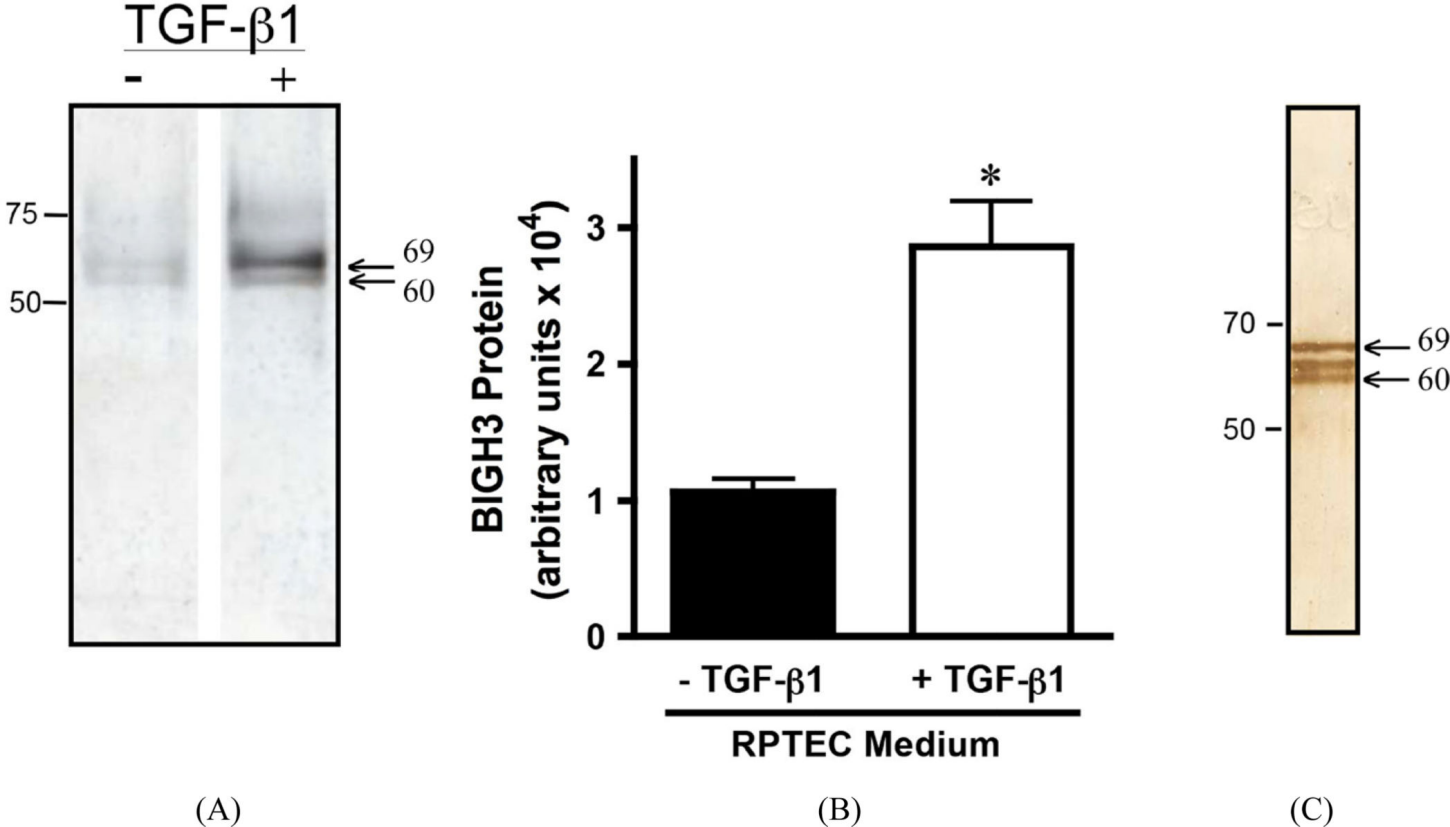
TGF-β1 upregulates BIGH3 protein expression in RPTEC. (A) Western blot of BIGH3 in the culture medium of RPTEC treated for 24 hours with vehicle only (−) or 42 pM TGF-β1 (+). (B) Densitometry of bands from 3 separate experiments quantified BIGH3 protein in (A), n = 3. Results are shown as mean ± S.D. The effect of TGF-β1 on increasing BIGH3 protein in the cells’ medium was significant, t-test (p < 0.05). (C) Western blot showing BIGH3 in the culture medium of a recombinant expression system (no TGF-β1 present). Arrows in (A) and (C) indicate BIGH3 protein bands. The 50-, 70- and 75-kDa protein standards are indicated. In each blot the lower band is consistent with the 60-kDa form of BIGH3 after C-terminal cleavage. The center protein band in (C) is the 62-kDa intermediate BIGH3 protein.

Treating RPTEC with 42 pM TGF-*β*1 for 24 hours more than doubled BIGH3 protein quantity in conditioned medium (Figure 5(A), Figure 5(B)). The TGF-*β*1-induced increase on renal cell BIGH3 synthesis is consistent with the MCM- and dMCM-induced increase on BIGH3 expression (Figure 3(A), Figure 3(B)). BIGH3 isolated from RPTEC conditioned medium was cleaved (Figure 5(A)). Interestingly, BIGH3 isolated from our Sf9 recombinant protein expression system was also cleaved (Figure 5(C)). Western blot staining resolved a 69-kDa protein, a 62 kDa C-terminus truncated intermediate BIGH3 [9], and a 60-kDa C-terminal truncated BIGH3 protein (Figure 5(C)). The three BIGH3 proteins coincide with mass spectrometry analysis, documenting BIGH3 C-terminal cleavage yields an intermediate 62-kDa BIGH3 protein and a relatively stable mature 60-kDa BIGH3 protein [7] [9] [31] and suggest that renal cells and other cell types cleave BIGH3 (see discussion). We conclude that TGF-*β*1 stimulates RPTEC to synthesize and secrete BIGH3, and that RPTEC cleave BIGH3 protein.

### BIGH3 promotes RPTEC apoptosis

**Figure 6:**
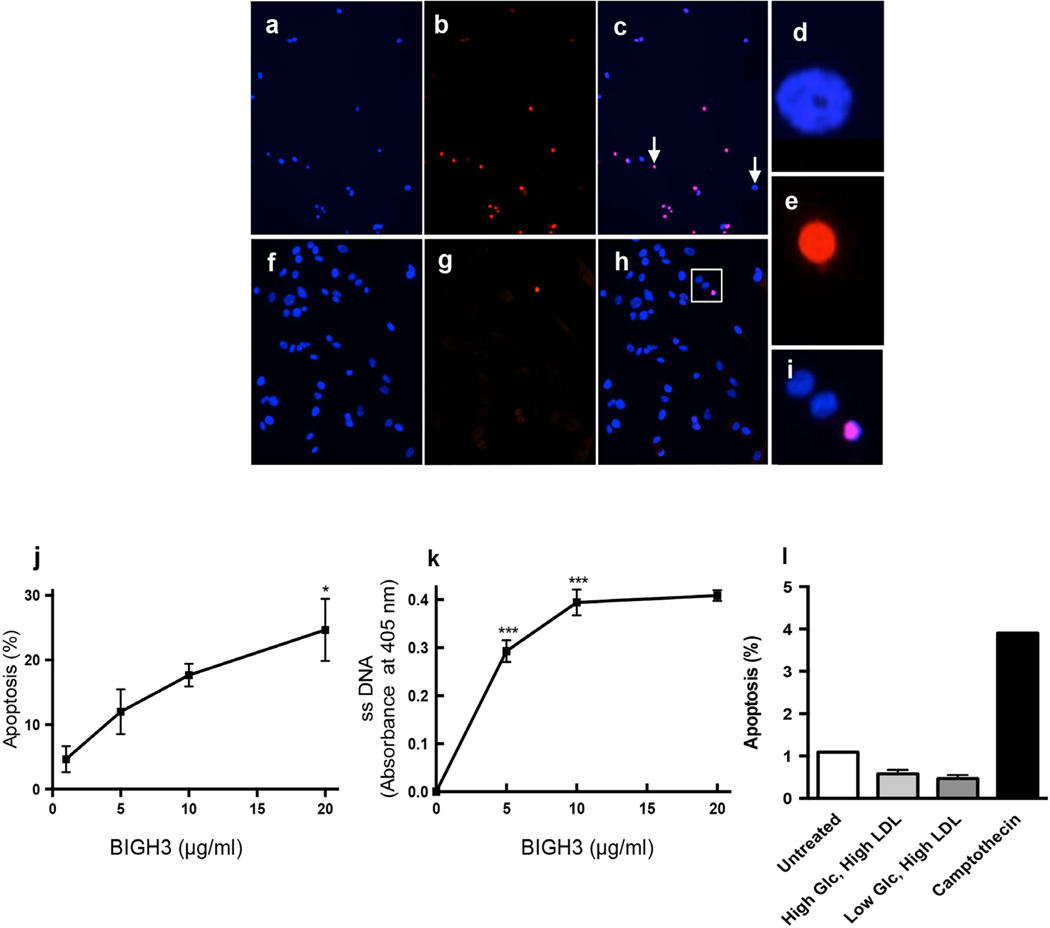
BIGH3 promotes renal cell apoptosis. RPTEC were treated with 5 µg/ml BIGH3 for 24 hours. (a) Cells stained with DAPI. (b) TUNEL labeled cells. (c) a and b merged. Images (a and b) were recorded using a 20× objective. Cell nuclei denoted by arrows in (c) were magnified using an identical digital command (no other parameters changed) to show non-apoptotic and apoptotic nuclei (d, e, respectively). Controls are cells treated with vehicle only, shown with (f) DAPI staining and (g) TUNEL labeling. (h) d and e merged. Images (g–h) were recorded using a 40× objective to better show nuclear condensation. (i) The boxed area in (h) is magnified to show apoptotic and non-apoptotic nuclei. (j) Cultured RPTEC were treated with increasing concentrations of rBIGH3 added directly to growth medium. After 24 hours, TUNEL labeling revealed a significant increase in BMA. Significance when comparing 20 µg/mL with 1 µg/mL (*p < 0.05) was observed by One Way ANOVA with a Newman-Keuls Multiple Comparison Test for significance between groups [F (3, 11) = 6.806; p < 0.05]. (k) ssDNA staining in a concentration response curve similarly show a significant increase in BMA of renal cells. The percent apoptosis and absorbance recorded in negative control wells (PBS-treated and non-treated wells) was subtracted from values shown in j and k. The One Way ANOVA for dose was significant [F (3, 35) = 105.1; p < 0.0001] (***p < 0.001). (l) TUNEL analysis of RPTEC cultured with 100 µg LDL in high and low glucose media show that, under conditions here, glucose and LDL did not increase cell apoptosis when compared to untreated cells in REGM.

Cleavage of BIGH3 C-terminus yields integrin ligand peptides that induce BMA in various cell types [8]–[12] [32]. To test for renal cell BMA, recombinant BIGH3 protein was added directly to REGM. After a 24-hour incubation period BMA was quantified using TUNEL assays, nuclei condensation analysis, and quantitation of single-stranded DNA. TUNEL and DAPI staining revealed an increase in the number of condensed nuclei in BIGH3-treated cells when compared to non-treated control cells (Figures 6(a)–(i)). The number of BIGH3-treated TUNEL-labeled cells was greater (Figure 6(b)) when compared to non-treated cells (Figure 6(g)). Non-apoptotic and apoptotic nuclei (Figure 6(c), arrows) were magnified to show nuclear condensation in cells in control REGM (Figure 6(d), Figure 6(e)). An apoptotic cell nucleus is compared to neighboring non-apoptotic cell nuclei (Figure 6(h), boxed area magnified in Figure 6(i)).

The extent of RPTEC BMA was dependent on the concentration of BIGH3 added to the growth medium. TUNEL and ssDNA assays quantified BMA of cells that were treated with 5 µg/ml BIGH3 (Figure 6(j), Figure 6(k), respectively). Increasing BIGH3 concentration to 10 µg/ml increased BMA, but there was no statistically significant change in the number of apoptotic cells when comparing effects of 10 and 20 µg/ml BIGH3. These results show that 5 µg/ml BIGH3 is sufficient to induce a significant increase in renal cell BMA. A 24-hour exposure to high glucose (25 mM) and LDL (100 µg/ml) (without exogenous dMCM, MCM and BIGH3) did not increase BMA of RPTEC when compared to untreated cells (Figure 6(l)) indicating that *in vitro* the signal promoting BMA is independent of glucose and LDL *per se*. We conclude that in addition to macrophage-derived injurious molecules (e.g., NO, ROS), macrophage-derived TGF-*β*1 and BIGH3 account for at least some of the renal cell damage and death in DN.

### BIGH3 is a key mediator of MCM and dMCM proapoptotic activity

**Figure 7:**
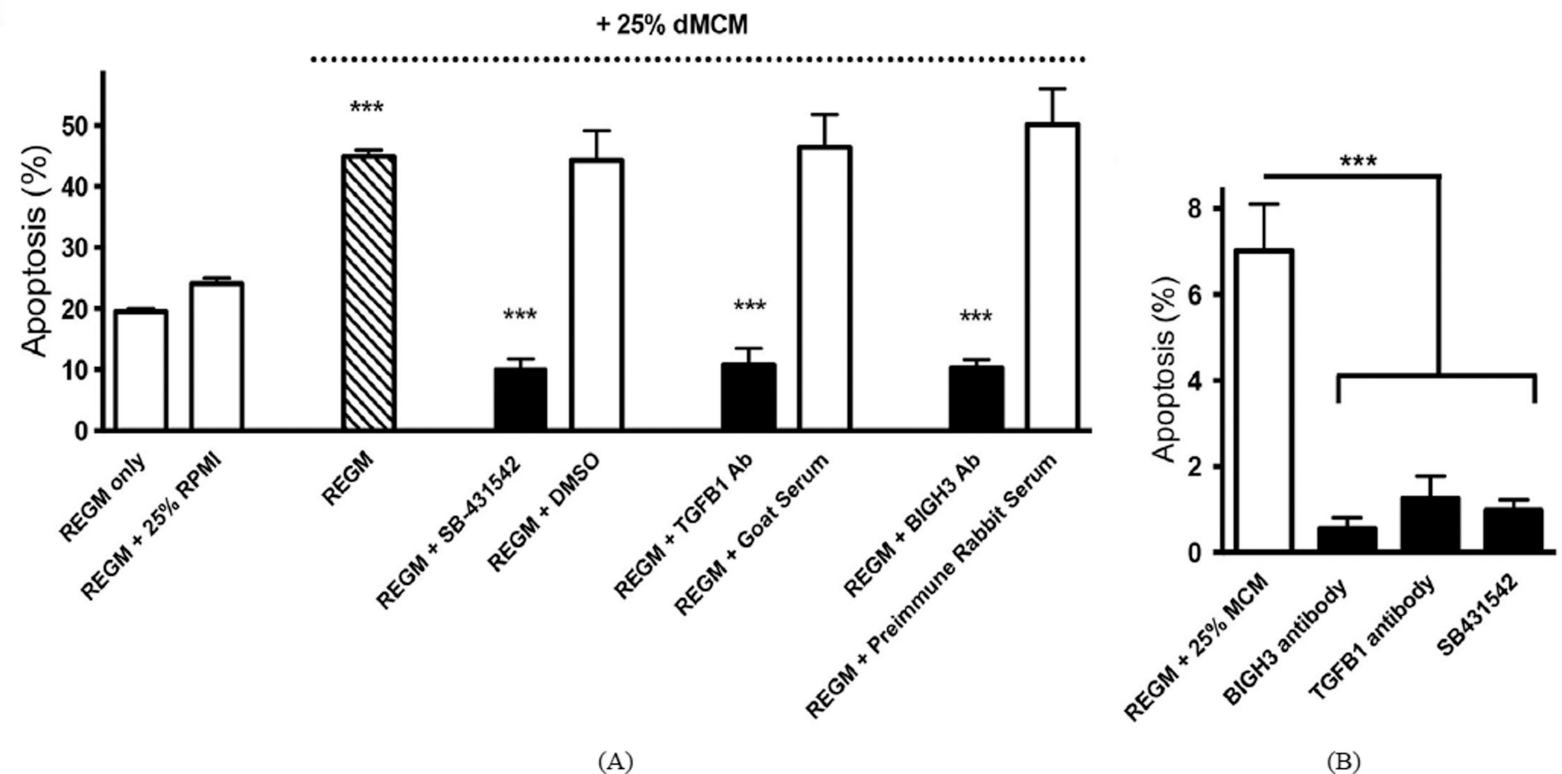
TGF-β1 inhibitors and BIGH3 antiserum block BMA. (A) Shown is BMA of RPTEC cultured in REGM + 25% dMCM with and without SB-431542, TGF-*β*1 Ab (goat anti-TGF-*β*1 antibody) and BIGH3 Ab (rabbit polyclonal anti-BIGH3 serum). Controls are vehicle only (DMSO), normal goat serum and preimmune rabbit serum. Three One Way ANOVAs were performed that included the three treatments and then each other treatment with its corresponding control. The effect of SB-431542 was significant [F (4, 14) = 4.31; p < 0.0001]. REGM + 25% dMCM was significantly different from either baseline controls (REGM only and REGM + 25% RPMI) at p < 0.001. REGM + SB-431542 was significantly different from REGM + 25% dMCM at p < 0.001. Vehicle DMSO had no effect. The effect of TGF-*β*1 antibody was significant [F (4, 14) = 32.7; p < 0.0001]. REGM + TGF-*β*1 Ab was significantly different from REGM + 25% dMCM at p < 0.001. Goat serum had no effect. The effect of BIGH3 antibody was significant [F (4, 14) = 38.77; p < 0.0001]. The REGM + BIGH3 Ab was significantly different from REGM + 25% dMCM at p < 0.001. Preimmune serum had no effect. Each comparison of REGM + SB-431542, REGM + TGF-*β*1 Ab, and REGM + BIGH3 Ab to REGM only was significant (p < 0.001). For each condition n ≥ 3 (***p < 0.001). (B) MCM induced BMA, which was blocked by BIGH3 antiserum, TGF-*β*1 antibody, and SB-431542. In REGM + 25% MCM the One Way ANOVA for SB-431542, TGF-*β*1 and BIGH3 Abs was significant F (3, 23) = 4.986, p ≤ 0.0001. For each condition n ≥ 3. Significance was set at p < 0.05.

RPTEC cultured in REGM plus 25% dMCM showed an 86% increase in BMA when compared to cells cultured in REGM plus 25% RPMI. To examine the percent apoptosis that TGF-*β*1 and BIGH3 induce, RPTEC were cultured in dMCM in the presence of TGF-*β*1-blocking and BIGH3-blocking agents (Figure 7(A)). A pharmacological inhibitor of TGF-*β* receptor activation (SB-431542), and anti-TGF-*β*1 antibody significantly blocked dMCM-induced BMA when compared to DMSO vehicle and serum controls. BIGH3 antiserum effectively blocked BMA when compared to preimmune serum. A significant increase in BMA was also evident in cells in MCM, in which BIGH3 antiserum, TGF-*β*1 antibody, and SB-431542 effectively and significantly blocked BMA (Figure 7(B)). We conclude that both MCM and dMCM promote apoptosis through a TGF-*β*1 and BIGH3-mediated mechanism.

## 4. Discussion

The results of this study agree with the principle that a hyperglycemic environment promotes macrophage infiltration and kidney damage. This report extends this principle, showing that macrophage-derived TGF-*β*1 promotes renal cell apoptosis by stimulating expression of the gene *TGFBI*, which encodes the extracellular matrix protein BIGH3. In the diabetic kidney cortex RPTEC are a probable source of BIGH3 [33]. BIGH3 in the cortical interstitial matrix is likely derived from resident mesenchymal cells. Macrophages and BIGH3 protein were broadly distributed in diabetic kidney cortices of our mouse model of diabetic complications [24]. However, immunological analysis unexpectedly revealed an increase in macrophage number, and BIGH3 protein, in the HFD dyslipidemic kidney when compared to non-diabetic kidney cortex. This result indicates a metabolically-stressed renal environment itself stimulates macrophage infiltration and BIGH3 protein expression, supporting the prospect that macrophage TGF-*β*1 and BIGH3 protein are prediabetic biomarkers. Indeed, others and we have reported that TGF-*β*1, and BIGH3, are at significantly higher levels in the urine of diabetic patients when compared to urine of non-diabetic individuals [34]–[36].

RPTEC in REGM control medium (without exogenous TGF-*β*1, BIGH3, or MCMs) expressed relatively low-levels of BIGH3 and exhibited BMA. BIGH3 antiserum and SB-431542 blocked the BMA observed in control medium, raising the possibility that in a healthy normoglycemic environment BIGH3 protein is involved in apoptosis in physiological cell turnover. In this perspective, healthy kidney cells synthesize a low level of new BIGH3 protein that induces modest, low-level BMA, which sustains cell turnover. Along this line of reasoning, this study supports the hypothesis that increased and accumulating BIGH3 protein in kidney cortex is an indicator of a prediabetic state [37]. Extending this view, a prediabetic environment promotes TGF-*β*1 signaling [38] BIGH3 synthesis, and BMA, and continues as disease progression develops DN.

It is well established that under physiological conditions BIGH3’s C-terminus is cleaved, generating C-terminal fragments less than 3 kDa that induce BMA [7]–[9] [39]. In contrast, a recombinant BIGH3 protein that did not undergo C-terminal cleavage failed to induce apoptosis [8], indicating that C-terminal cleavage is a requisite for BMA. C-terminally cleaved BIGH3 protein has been found *in vivo* in cornea, skeletal muscle and tendon [7] [27]. The peptidases serine protease high-temperature requirement A1 [40], plasmin [31], and matrix metalloproteinase 9 [41] have been implicated in BIGH3 cleavage. However, the extent of cleavage, and which proteases cleave kidney cortical BIGH3 *in vivo*, in healthy tissue, and in metabolically stressed conditions, remains unclear.

We show here that TGF-*β*1-treated and untreated RPTEC cleave endogenous BIGH3, and thus these cells would be expected to cleave exogenous recombinant BIGH3, explaining the increase in BMA in BIGH3 protein add-back experiments. However we also note that some BIGH3 is cleaved when isolated from Sf9 medium, indicating Sf9 cells cleave BIGH3. Therefore the apoptosis quantified in add-back experiments may have been induced from BIGH3 already cleaved by Sf9 cells (recombinant BIGH3 containing uncleaved and cleaved BIGH3 that was used in add back experiments) and also by RPTEC cleaving the uncleaved BIGH3 recombinant protein that was added back. Mass spectrometry and biochemical analysis revealed C-terminal cleavage accounts for lower mass BIGH3 proteins [7] [9] [31], which we have previously shown to migrate on Western blots as a 62-kDa intermediate band and a stable 60-kDa lower band [9]. C-terminal cleavage generates peptides that likely comprise the integrin-binding sequences EPDIM (residues 615 – 619) and RGD (642 – 644). Both of these integrin-binding sequences have been implicated in BMA, and introduced point mutations in EPDIM and RGD have blocked BMA in various different cell types [8]–[10] [12].

The data presented in this study frame a potential feedback mechanism. Firstly, injured kidney cortex recruits monocyte-derived macrophages. These macrophages provide TGF-*β*1 that stimulates RPTEC to synthesize and secrete BIGH3. Secondly, C-terminal cleavage generates integrin ligand peptides. In a paracrine or autocrine mechanism the peptides induce BMA, presumably involving integrins. Thirdly, an increase in the number of apoptotic cells promote phagocytic cell infiltration, increasing macrophage number, macrophage-derived TGF-*β*1, and BMA. Macrophage ingestion of apoptotic bodies induce macrophages to release additional TGF-*β*1 [19] and moreover, stimulates macrophages themselves to synthesize and secrete BIGH3 protein [42]. RNA-Seq data from TGF-*β*1 stimulated human kidney epithelial cells compared with microarray data from human DN renal biopsies revealed 179 genes presumed to be upregulated by TGF-*β*1 signaling. The BIGH3 gene was the most significantly upregulated gene in a TGF-*β*1 induced transcriptional profile [43]. In addition to RPTEC BMA shown here, evidence suggests that BIGH3 negatively regulates E-cadherin transcription and translation in human renal epithelial cells [43] and dissociate VE-cadherin junctions between endothelial cells [43] [44], thereby promoting epithelial myofibroblast transdifferentiation (EMT) and providing myofibroblast-derived TGF-*β*1 [45] [46]. Interestingly, integrins *α*v*β*3, *α*v*β*5, *α*M*β*2, and *α*3*β*1 bind sequence in the FAS1-like domains of the relatively stable 60-kDa BIGH3 protein [5] [47]–[49] raising the possibility that *in vivo* the 60-kDa BIGH3 protein is a macrophage adhesion substrate, potentiating a robust, self-propagating positive feedback stimulus of a mechanism propelled by a metabolically-stressed environment, macrophage-derived TGF-*β*1, BIGH3 synthesis, EMT and BMA.

## 5. Conclusion

This study describes a novel mechanism underlying TGF-*β*1-induced apoptosis *i.e.* BIGH3 synthesis and cleavage. The results distinguish macrophage-derived TGF-*β*1 and BIGH3 as potential biomarkers, and targets for prediabetic and diabetic therapeutic interventions. We have recently reported that macrophages infiltrate diabetic human retina, and that our MCMs induced BMA of retinal endothelial cells, implicating BMA in retinal completions in patients with poorly-controlled diabetes [11]. Others have shown that BMA targets retinal pericytes [10], which we have confirmed and show involvement of a similar mechanism leading to an increase in BMA (manuscript in preparation). These findings substantiate the probability that BMA plays a significant role promoting microvascular and macrovascular diseases, underlining the importance of regulating genes encoding ECM molecules.
